# Mendel’s legacy in modern genetics

**DOI:** 10.1371/journal.pbio.3001760

**Published:** 2022-07-28

**Authors:** Joanna Clarke, on behalf of the PLOS Biology Staff Editors

**Affiliations:** Public Library of Science, San Francisco, California, United States of America and Cambridge, United Kingdom

## Abstract

A new collection of articles celebrating the bicentennial of Gregor Mendel’s birth discuss his life, work and legacy in modern-day genetic research

The field of biology owes a great debt to both genetic material and those who study it. From tiny bacteria to colossal giant sequoias, genetic material is the common thread that runs through all life forms and is even found in infectious agents such as viruses and in transposable elements. As such, although a field of study in its own right, genetics underpins every branch of biology and forms an important part of the majority of research questions.

July 20^th^ 2022 marked the 200^th^ anniversary of the birth of the scientist-monk J. Gregor Mendel, widely regarded as the founder of genetics. His experiments in selectively breeding pea plants and observing the way that different traits were passed on to each generation [1] paved the way for our current understanding of the principles that govern inheritance and have influenced present-day applications of genetic research, from *in utero* testing for genetically inherited diseases to genetically engineering crops to increase yields or nutritional content. Moreover, his meticulous approach to gathering and recording data is presented to biology students as a textbook example of the scientific method in practice.

In recognition of the legacy and impact of Mendel’s ground-breaking work, *PLOS Biology* has assembled a special collection of articles on the theme of ‘Mendel’s legacy in modern genetics’. In the collection, you will find Perspective articles from experts working across the field of genetics on how Mendel’s work has shaped their areas of interest [2,3,4], as well as an exploration of Mendel’s life and work as a scientist told through his own words [5]. The collection also contains Essays exploring different aspects and applications of modern genetics research; Sarah Garland and Helen Anne Curry use historical perspectives to ask whether gene editing of crops has lived up to its potential, charting the process from its early beginnings in Mendel’s work [6], and Laurence Hurst asks whether a greater understanding of selfish genetic elements, which do not adhere to the principles of Mendelian inheritance, can explain why so many human embryos have the wrong number of chromosomes and fail to develop [7].

The collection will be updated with content throughout the year, and we hope that you will take inspiration from our look back at Mendel’s original research and examination of how well it stands up to modern views on genetics. In the words of Eva Matalova in her sketch of Mendel’s life [5], “[t]o the present day, his ideas, modern attitude, and way of scientific critical thinking made his legacy ever living”.
